# Evaluation of the trends in the incidence of infectious diseases using the syndromic surveillance system, early warning and response unit, Mongolia, from 2009 to 2017: a retrospective descriptive multi-year analytical study

**DOI:** 10.1186/s12879-019-4362-z

**Published:** 2019-08-09

**Authors:** Badral Davgasuren, Suvdmaa Nyam, Tsoggerel Altangerel, Oyunbileg Ishdorj, Ambaselmaa Amarjargal, Jun Yong Choi

**Affiliations:** 1 Graduate School of Public Health, Yonsei University, Seoul, South Korea; 2 Department of Surveillance and Prevention of Infectious diseases, National Center for Communicable Diseases, Ulaanbaatar, Mongolia; 3 Department of Internal Medicine and AIDS Research Institute, Yonsei University College of Medicine, Seoul, South Korea

**Keywords:** Infectious diseases syndrome, Syndromic surveillance system, Mongolia

## Abstract

**Background:**

In recent times, emerging and re-emerging infectious diseases are posing a public health threat in developing countries, and vigilant surveillance is necessary to prepare against these threats. Analyses of multi-year comprehensive infectious disease syndrome data are required in Mongolia, but have not been conducted till date. This study aimed to describe the trends in the incidence of infectious disease syndromes in Mongolia during 2009–2017 using a nationwide syndrome surveillance system for infectious diseases established in 2009.

**Methods:**

We analyzed time trends using monthly data on the incidence of infectious disease syndromes such as acute fever with rash (AFR), acute fever with vesicular rash (AFVR), acute jaundice (AJ), acute watery diarrhea (AWD), acute bloody diarrhea (ABD), foodborne disease (FD) and nosocomial infection (NI) reported from January 1, 2009 to December 31, 2017. Time series forecasting models based on the data up to 2017 estimated the future trends in the incidence of syndromes up to December 2020.

**Results:**

During the study, the overall prevalence of infectious disease syndromes was 71.8/10,000 population nationwide. The average number of reported infectious disease syndromes was 14,519 (5229-55,132) per year. The major types were AFR (38.7%), AFVR (31.7%), AJ (13.9%), ABD (10.2%), and AWD (1.8%), accounting for 96.4% of all reported syndromes. The most prevalent syndromes were AJ between 2009 and 2012 (59.5–48.7%), AFVR between 2013 and 2014 (54.5–59%), AFR between 2015 and 2016 (67.6–65.9%), and AFVR in 2017 (62.2%). There were increases in the prevalence of AFR, with the monthly number of cases being 37.7 ± 6.1 during 2015–2016; this could be related to the measles outbreak in Mongolia during that period. The AFVR incidence rate showed winter’s multiplicative seasonal fluctuations with a peak of 10.6 ± 2 cases per 10,000 population in 2017. AJ outbreaks were identified in 2010, 2011, and 2012, and these could be associated with hepatitis A outbreaks. Prospective time series forecasting showed increasing trends in the rates of AFVR and ABD.

**Conclusions:**

The evidence-based method for infectious disease syndromes was useful in gaining an understanding of the current situation, and predicting the future trends of various infectious diseases in Mongolia.

## Background

In the last two decades, although the burden associated with communicable diseases has been reducing through deliberated efforts, worldwide, emerging and re-emerging infectious diseases are still public health threats in developing countries due to globalization. There is a need for constant readiness and preparedness to deal with infectious disease outbreaks including emerging and re-emerging infectious disease threats.

Public health risks can be detected using the syndromic surveillance system (SSS) that includes event-based surveillance. The SSS has been implemented since the 1990s and its initial purpose was bioterrorism detection. In the United States (US), SSS use became widespread in early 2000 [1, 2]. The Centers for Diseases Control and Prevention defined syndromic surveillance (SS) as “surveillance using health-related data that precede diagnosis and signal a sufficient probability of a case or an outbreak to warrant further public health response” [3]. Surveillance is the ongoing systematic collection, analysis, interpretation, and application of real-time indicators that allows for the detection of diseases before they would otherwise be identified by public health authorities. SS in public health surveillance emphasizes the use of near real-time, pre-diagnostic data and statistical tools to detect and characterize unusual activity for further public health investigation. The goal of syndrome surveillance is the routine gathering of electronic health data and earlier anomaly detection of epidemics, allowing for a timelier public health response utilizing pre-diagnostic data sources than is possible using conventional surveillance methods, and the monitoring of illness progression in a population [1, 2, 4–7]. SS offers superior timeliness and flexibility compared to diagnosis-based surveillance. However, as SS is a relatively new approach, there is still a lack of clarity on its added value.

Mongolia has a comprehensive surveillance system for infectious disease (ID) consisting of a national indicator-based, syndromic, event-based, and many disease-specific surveillance systems managed by both the National Center for Communicable Diseases (NCCD) and National Center for Zoonotic Diseases. There are 55 types of notifiable infectious diseases. Surveillance data from each system are regularly reported, with most being shared at weekly inter-sector surveillance meetings, and distributed through the NCCD email distribution list and published on the NCCD website.

Mongolian health services are provided at primary, secondary and tertiary health care facilities across two administrative divisions (the capital and the provinces). The Mongolian health system is heavily a hospital-oriented system. Hence, any disease’s information system in spans three levels: all family health centers at the primary level inform health centers in sub-provinces and public health centers in nine districts at the secondary level nationwide. Secondary information flows from health centers in 329 sub-provinces, and public health centers and general hospitals in districts report to health departments in 21 provinces and in the capital city. Health care facilities at the secondary level report to the NCCD and National Center for Zoonotic Diseases.

The Mongolian SSS for infectious diseases was established under the Early Warning and Response (EWAR) unit of the Department of Surveillance and Prevention of Infectious Disease at the NCCD for the early detection of public health threats and outbreaks, for tracking infectious disease syndromes, and for promptly responding to events if necessary nationwide. It was initially piloted in three provinces in 2007 and then expanded throughout the entire country, including all 21 provinces and nine districts in the capital city for 2 years until 2009. The SSS consists of case-based surveillance and was integrated with an event-based surveillance system in 2010. Surveillance units work in health centers, public health centers, and health departments in all provinces and districts in the capital city at the primary and secondary levels, and the national surveillance unit for SSS works under the EWAR unit at the NCCD. The SSS in the EWAR unit also has three reporting levels similar to the information system for other infectious diseases. Physicians and health workers in all 1681 family health centers at the primary level receive data on patient visits, report diagnoses of the patients, and electronically inform 363 Health centers in sub-provinces and districts at the secondary level via specialized information forms for ID syndromes according to case definitions for ID syndromes, suspected cases, and special events every Monday. Surveillance units at the secondary level receive this information and combine, analyze the data, and report to tertiary centers.

SSS collects data on infectious disease syndrome type, reporting date, demographic information, onset of symptoms, admission date, and previous diagnosis of every suspected case. The health centers in sub-provinces and districts inform health departments in the 21 provinces and the capital city and electronically transmit reports via EWAR 2.0 software every Tuesday to secondary level surveillances unit for infectious disease syndrome. The tertiary level units of the EWAR unit combine data from the EWAR 2.0 software, conduct descriptive data analyses, develop conclusions and responses in accordance with the conditions, and send feedback on the ID syndromes, suspected cases, and special events to all reporting health facilities at the primary and secondary level, as well as other related organizations, including the Ministry of Health (MoH), every Thursday via email. For some reports, such as those on foodborne diseases (FDs), reporting within 2–24 h by calling is required. Weekly feedback from descriptive analysis at the tertiary level includes only information on the numbers of each ID syndrome, suspected cases, and special events by province, district, and age group.

Case definitions of 22 ID syndromes, suspected cases and special events have been standardized and approved by order No. 152, 2010 of the Health Minister. The subject of the SSS in the EWAR unit includes 11 infectious disease syndromes: acute flaccid paralysis (AFP), acute fever with rash (AFR), acute fever with vesicular rash (AFVR), acute jaundice (AJ), acute watery diarrhea (AWD), acute bloody diarrhea (ABD), acute respiratory infection (ARI), influenza and influenza-like illness (ILI), acute lower respiratory infection (ALRI), acute hemorrhagic syndrome (AHS), acute neurologic syndrome (ANS); seven suspected cases: tetanus, neonatal tetanus, pertussis, diphtheria, anthrax, plague and rabies; and four special events: FDs, nosocomial infections (NIs), adverse events following immunization and unexplained clusters of health events.

Currently, large amounts of infectious disease data are routinely collected by laboratories, healthcare providers and government agencies in an effort to increase the understanding of their evolution and prevent, detect, and manage infectious disease outbreaks. Due to the emergence and re-emergence of infectious diseases with pandemic potential, over the past decade, there has been a surge in interest on the associated analysis and statistical methods. This increase in interest has given rise to new methodological work, ranging across the spectrum of statistical methods for the early detection of infectious disease outbreaks. Therefore, there is an urgent need to monitor and predict these syndromes for effective outbreak control. In this context, one-step-ahead forecasts, especially when syndrome information is incorporated into the forecasting model, can be used to detect high-risk areas for outbreaks and, consequently, to develop effective targeted surveillance [8]. Accurate and reliable disease forecasting can be of tremendous value to public health. However, analyses of multi-year comprehensive infectious disease syndrome data have not been conducted till date. We can be use number of suspected cases of detailed certain infectious diseases of each syndromes to early prevent and detect the outbreaks and epidemics based this information.

The current study was performed with the aim of describing the trends in the incidence of infectious diseases using the SSS of the EWAR unit in Mongolia from January 2009 to December 2017. In addition, we assessed the magnitude change in the occurrence frequencies of these diseases and forecasted future incidence trends.

## Methods

### Study design

This retrospective descriptive multi-year analytical study utilized data on infectious disease syndromes from the EWAR unit, as reported from January 1, 2009 to December 31, 2017. This study was part of the departmental data review process in the EWAR unit of the Department of Surveillance and Prevention of Infectious Disease at the NCCD. Although no personal identifiers were involved and no individuals stood to be jeopardized by the study, institutional research guidelines following the study approval were adhered to ensure scientific soundness.

### Collected data

As this study analyzed syndromic surveillance data from all family health centers in 363 sub-provinces and sub-districts at the primary level that were transmitted through health departments and general hospitals in 21 provinces and public health centers and general hospitals in nine districts in the capital city, as well as syndromic surveillance data collected from tertiary state central hospitals, specialized centers, regional diagnostic and treatment centers, maternal hospitals, and other large private hospitals at the tertiary level, all located in the capital city. The primary and secondary levels surveillance units such as public health facilities (84%) and private hospitals (16%) that are a part of the national surveillance system for infectious disease syndrome, the data are representative of the national syndromic surveillance situation, subject to reporting rates from all health institutions; therefore, no sample size estimates are required.

Data were collected from national surveillance system through the EWAR 2.0 electronic database weekly feedback, which is sent to all surveillance units including all family health centers, health departments and public and private hospitals in 21 provinces and 9 districts in Ulaanbaatar city. It includes the number of cases for each of the eleven ID syndromes, seven suspected cases*,* and four special events.

For this study, data on the five most prevalent infectious disease syndromes: AFR, AFVR, AJ, AWD, ABD and two special events (FD and NI) of the 22 infectious disease syndromes were analyzed. Data pertaining to two types of ARI syndromes: ARI influenza and ILI, and ALRI were excluded because they have been reported separately using the sentinel surveillance system since 2009, so there may be overlaps and underreporting in the SSS. In addition, data on rare events with an incidence < 1/10,000 population per year such as in the case of AFP, AHS and ANS, the seven suspected cases and two special events were not analyzed.

### Statistical analyses

Time trend analysis for incidence involved the following seven prevalent infectious disease syndromes: AFR, AFVR, AJ, AWD, ABD syndromes, and two special events (i.e., FD and NI). Incidence was calculated from the total number of syndromes of infectious diseases divided by the total of the specific population multiplied by 10,000. Time trend in the annual incidence of infectious disease syndromes were assessed by Poisson log-linear regression model with scaled standard errors to deal with overdispersion. Year was considered independent variable, with annual incidence rate as dependent variables. We conducted time series forecasting considering seasonality. We detected trend and seasonality of the time series by visualizing the incidence rate of syndrome over time and using autocorrelation function (ACF). As a result, we found that AFVR, AJ, AWD, ABD, FD, and NI had seasonality, and they were predicted considering seasonality. Prospective time series forecasting in the incidences of infectious disease syndromes was conducted by Auto-Regressive Integrated Moving Average (ARIMA) models or additive seasonal exponential smoothing method until December 2020.

Before fitting models, if the variance of incidence rate data was not stationary over time, the variance was stabilized by log transformation. For forecasting AFR, AFVR, AJ, AQD and ABD, seasonal ARIMA models were applied in accordance with Box-Jenkins approach, which consist of three steps: model identification, parameter estimation, and model checking. Seasonal ARIMA model was identified with ACF plot, PACF plot, and Akaike information criterion. Portmanteau test was used for checking autocorrelation in residuals. Statistical analyses were performed using SAS (version 9.4, SAS Inc., Cary, NC, USA) and R package, version 3.4.4 (The R Foundation for Statistical Computing, Vienna, Austria). Statistical significance was set as *p* < 0.05.

To test the rate trend per year, Poisson regression model was estimated. In Poisson regression model, incidence rates were calculated as numbers per unit time given by the formula: Explanatory variable Log(E(Y/x)) = α + βx, where Y is the average rate of events for a given time (dependent variable) and α is the mean of Y, β-predictor value, and x - predictor variables (independent variable). In our study, dependent variable was the annual incidence rate, and independent variable was year.

Forecasting future trends in the incidences of syndromes based on the data up to 2017 were assessed until December 2020 using SAS (version 9.4).

## Results

### Incidence and prevalence of infectious disease syndromes

The nationwide overall prevalence of all infectious disease syndromes was 71.8 per 10,000 population between 2009 and 2017. The average number of all infectious disease syndromes was 14,519 (range 5229 to 55,132) per year. The five most prevalent infectious disease syndromes, accounting for 96.4% of all the reported cases were: AFR (38.7%), AFVR (31.7%), AJ (13.9%), ABD (10.2%), and AWD (1.8%). The average number of cases with the seven major syndromes was 14,687 (range 4070 to 54,522) per year. The average incidence rate of the seven major infectious disease syndromes was 9.2 (0.04–34.5) per 10,000 population.

The incidence of AFR was the highest in 2016 (49.4%, range 36,256/73,393), and lowest in 2009 (0.2%, range 163/73,393). The incidence of AFVR was the highest in 2017 (32.7%, range 19,659/60,082) and lowest in 2009 (0.9%, range 551/60,082). The highest prevalence of AJ was reported in 2011 (32%, range 8441/26,383) while the lowest was recorded in 2016 (2.5%, range 668/26,383). The highest prevalence of AWD was reported in 2017 (16.7%, range 585/3507) while the lowest was recorded in 2011 (7.7%, range 271/3507). The highest prevalence of ABD was reported in 2017 (25.8%, range 5028/19,476) while the lowest was recorded in 2009 (3%, range 593/19,476). The highest prevalence of FD was reported in 2016 (25.5%, range 427/1675) while the lowest was recorded in 2009 (1.4%, range 23/1675). The highest prevalence of NI was reported in 2011 (19.2%, range 64/333) while the lowest was recorded in 2009 (4.8%, range 16/333).

Poisson regression analyses showed statistically significant increase of incidence rate for AFR (*p* = 0.0162), AFVR (*p* < 0.001), ABD (*p* < 0.001), and FD (*p* = 0.0015). Incidence rate ratios (95% CI) for AFR, AFVR, ABD, and FD were 1.599 (1.091, 2.344), 1.459 (1.393, 1.528), 1.288 (1.217, 1.363), and 1.201 (1.072, 1.344), respectively. Incidence rates of AJ, AWD, and NI were not significantly changed (Table 1).

**Table 1:** Annual trends in the incidence of major infectious disease syndromes by year in Mongolia, 2009–2017

Syndromes	Annual incidence and proportion, cases/10,000 population (%)									*P* value
	2009	2010	2011	2012	2013	2014	2015	2016	2017	
AFR	0.6(4)	1.1(3.8)	1.8 (4.1)	3.2 (8.8)	2.8 (7.5)	3.6 (9.6)	91.7 (67.6)	116.2 (65.9)	17.1 (16.8)	0.0162
AFVR	2 (13.4)	3.7 (12.9)	7 (16.3)	7.9 (21.5)	20.1 (54.5)	22.2 (59)	26.9 (19.9)	44.3 (25.1)	63 (62.2)	< 0.001
AJ	9 (59.5)	17.4 (60.3)	30 (69.6)	17.8 (48.7)	6.9 (18.7)	3.7 (9.9)	3.5 (2.6)	2.1 (1.2)	2.2 (2.2)	0.1416
AWD	1.2 (7.7)	1.8 (6.4)	0.9 (2.2)	1.3 (3.6)	0.9 (2.5)	1.1 (2.9)	1.6 (1.2)	1.2 (10.7)	1.9 (1.9)	0.143
ABD	2.2 (14.4)	4 (13.9)	2.7 (6.2)	5.9 (16.2)	5.6 (15.1)	6.2 (16.5)	11.1 (8.2)	10.9 (6.2)	16.1 (15.9)	< 0.001
FD	0.1 (0.6)	0.7 (2.4)	0.4 (1.0)	0.4 (1)	0.5 (1.4)	0.7 (1.8)	0.7 (0.5)	1.4 (0.8)	0.8 (0.8)	0.0015
NI	0.06 (0.4)	0.07 (0.2)	0.23 (0.5)	0.12 (0.3)	0.16 (0.4)	0.1 (0.3)	0.1 (0.1)	0.15 (0.1)	0.14 (0.1)	0.1995

*AFR* acute fever with rash, *AFVR* acute fever with vesicular rash, *AJ* acute jaundice, *AWD* acute watery diarrhea, *ABD* acute bloody diarrhea, *FD* foodborne disease, *NI* nosocomial infection

*P*-values are tested for trend test. Incidences are calculated by cases /10,000 population, and proportions are expressed as % of the syndrome among total reported cases. Underlined text shows the highest incidence of the year

Table 1 shows the annual incidence r1ates of the seven syndromes between 2009 and 2017. The most prevalent syndromes were AJ between 2009 and 2012 (59.5–48.7%), AFVR between 2013 and 2014 (54.5–59%), AFR during 2015 and 2016 (67.6–65.9%), and AFVR in 2017 (62.2%), as shown in Table 1.

### Time series analysis and forecasting for infectious diseases syndromes

Forecasting models for AFR, AFVR, AJ, AWD, and ABD were ARIMA(1,0,1), ARIMA(0,1,1,)(0,1,1)_12_, ARIMA(0,1,1)(0,1,1)_12_, ARIMA(1,1,1)(1,1,1)_12_, ARIMA(1,1,1)(1,1,1)_12_, respectively (Table 2). For forecasting FD and NI, additive seasonal exponential smoothing methods were applied.

**Table 2:** Forecasting models for infectious diseases syndromes in this study

Infectious diseases syndromes	Forecasting models
AFR	ARIMA(1,0,1)
AFVR	ARIMA(0,1,1,)(0,1,1)_12_
AJ	ARIMA(0,1,1)(0,1,1)_12_
AWD	ARIMA(1,1,1)(1,1,1)_12_
ABD	ARIMA(1,1,1)(1,1,1)_12_
FD	Additive seasonal exponential smoothing method
NI	Additive seasonal exponential smoothing method

*AFR* acute fever with rash, *AFVR* acute fever with vesicular rash, *AJ* acute jaundice, *AWD* acute watery diarrhea, *ABD* acute bloody diarrhea, *FD* foodborne disease, *NI* nosocomial infection, *ARIMA* Auto-Regressive Integrated Moving Average

**Fig. 1:**
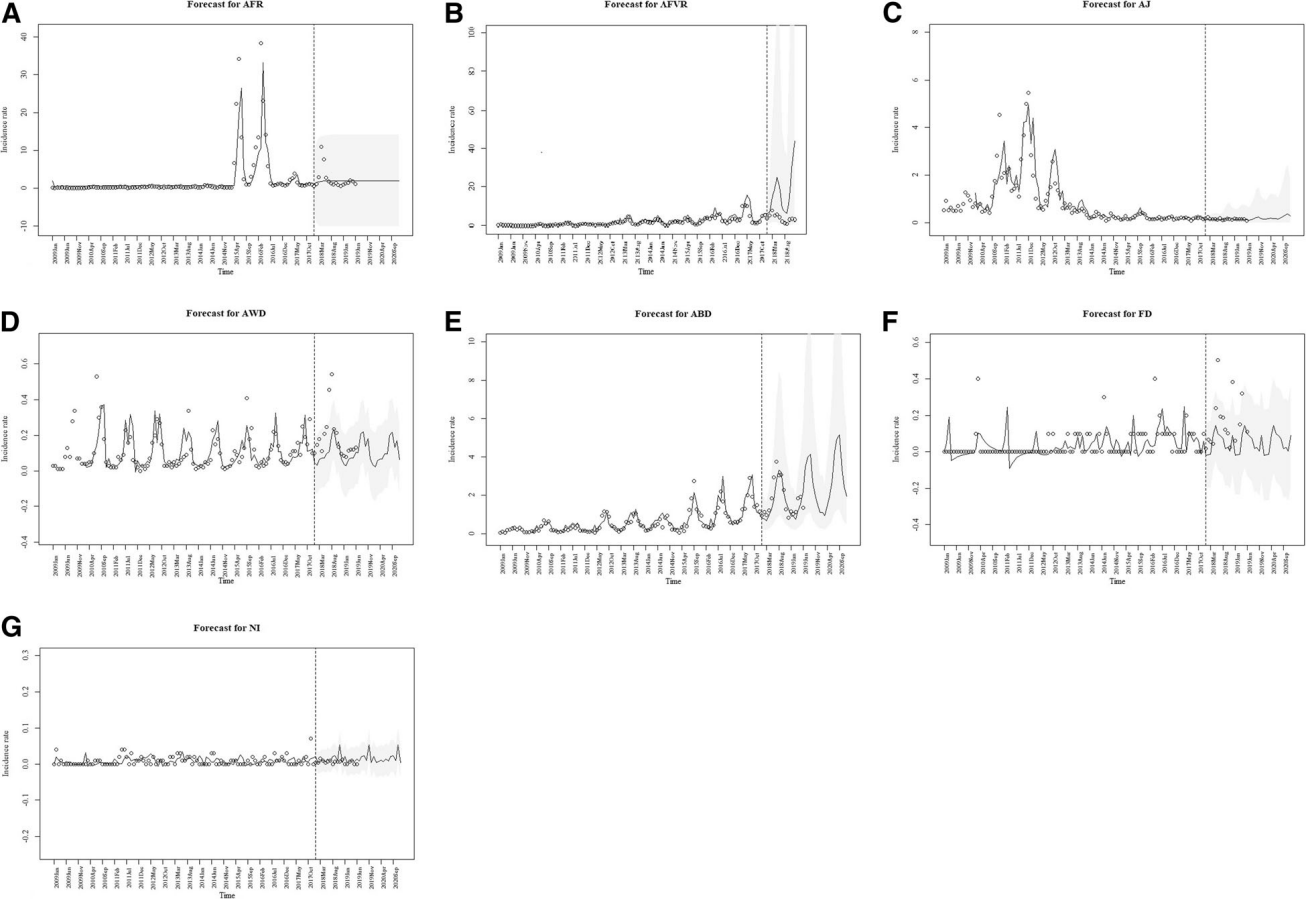
Time series analysis of the monthly number of reported cases with infectious disease syndromes in Mongolia from Jan 2009 to Dec 2017 and forecasting the trends up to Dec 2020. **a**. Acute fever with rash **b**. Acute fever with vesicular rash **c**. Acute jaundice **d**. Acute watery diarrhea **e**. Acute bloody diarrhea **f**. Foodborne disease **g**. Nosocomial infection**.** AFR, acute fever with rash; AFVR, acute fever with vesicular rash; AJ, acute jaundice; AWD, acute watery diarrhea; ABD, acute bloody diarrhea; FD, foodborne disease; NI, nosocomial infection **.** Horizontal axis is the month for measurement, and vertical axis is the incidence rate of reported cases by 10,000 population, as indicated. Forecasting models for AFR, AFVR, AJ, AQD, ABD, FD, and NI are ARIMA(1,0,1), ARIMA(0,1,1,)(0,1,1)_12_, ARIMA(0,1,1)(0,1,1)_12_, ARIMA(1,1,1)(1,1,1)_12_, ARIMA(1,1,1)(1,1,1)_12_, additive seasonal exponential smoothing method, and additive seasonal exponential smoothing method, respectively. Circles are actual data, and solid line shows forecasted data. Dotted lines indicates the time point at which the observed (left of the dotted line) and predicted (right of the dotted line) values are divided. Gray zone shows the 95% confidence limits.. The forecast for AFVR was assessed until the end of 2018, because the forecast for AFVR was too sensitive to the increasing trend near the end of 2017, and resulted in unreasonable forecast in long-term forecasting

Figure 1 presents the monthly time series data for infectious diseases syndromes in Mongolia from 2009 to 2017.

The highest prevalence rate (37.7 ± 6.1 cases monthly) of AFR syndrome in 2015–2016 could be related to the measles outbreak in Mongolia during 2015–2016. According to currently available data, there is a likelihood that the outbreak started before March 2015, so the general timeline trend in the incidence of AFR syndrome for 2009–2017 was significantly increased during the study period and prospective time series forecasting predicted a continuous stable trend in future years (Fig. 1a).

The incidence rate of AFVR showed winter’s multiplicative seasonal fluctuations with a peak at 10.6 ± 2 cases per 10,000 people in 2017. The highest incidence was observed during autumn and spring every year. The general timeline trend in the incidence of AFVR may have risen significantly over the study period, with prospective time series forecasting showing a continuous rising trend in future years (Fig. 1b). The forecast for AFVR was assessed until the end of 2018, because the forecast for AFVR was too sensitive to the increasing trend near the end of 2017, and resulted in unreasonable forecast in long-term forecasting.

There were outbreaks related to AJ between 2010 and 2012, most likely due to hepatitis A outbreaks. The highest incidence of AJ was 5.5 ± 1 per 10,000 population in the Winter’s multiplicative model. The general timeline trend in the incidence of AJ did not show significant change over the study period, while prospective time series forecasting showed a continuous decreasing trend in future years (Fig. 1c).

The AWD incidence rate showed simple seasonality and roughly seasonal fluctuations from spring to autumn, particularly May to October, with a peak of 0.5 ± 0.1 cases per 10,000 population in 2010. General timeline trends in the incidence of the syndrome were consistent during the study period, with prospective time series forecasting showing continuous consistency with seasonality in future years (Fig. 1d). This syndrome was found to have simple seasonality, and roughly seasonal fluctuations that slowly increase in spring and decrease in autumn every year, particularly from May to October, with a peak of 2.9 ± 0.6 cases per 10,000 population.

General timeline trends in the incidence of ABD showed a slight annual increasing trend during the study period, and prospective time series forecasting showed a continuous rising trend in future years (Fig. 1e).

The incidence rate of FD showed simple seasonality with irregular fluctuations, with a peak of 0.4 ± 0.1 cases per 10,000 population in 2017. The highest incidence rates of FD cases were reported in 2010, 2014, and 2016. General timeline trends showed significant rising trend during the study period, and prospective time series forecasting showed a consistency with seasonality in future years (Fig. 1f).

The incidence rate of NI showed simple seasonality with irregular fluctuations, and a peak of 0.07 cases per 10,000 population. The highest incidence rates were observed in 2009, 2011 and 2017, with the highest number of cases being reported in 2017. General timeline trends showed no difference trend in the incidence of NI during last 9 years, with prospective time series forecasting showing continuous consistency in future years (Fig. 1g).

The observed data of 2018 and 2019 are also presented within Fig. 1. For AFR, AFVR, AJ, ABD, and NI, the observed incidences were within the 95% confidence limits of forecasted data. For AWD and FD, there were some sporadic time points with observed incidences higher than 95% confidence limits of forecasted data.

## Discussion

This study assessed the trends in the incidence of infectious disease syndromes using an SSS, from 2009 to 2017, in Mongolia, and performed probabilistic time series forecasting of infectious disease syndromes. Retrospective time series analysis showed positive trends in the incidence of AFR, AFVR, ABD, and FD. An assessment was performed using winters’ multiplicative seasonality model for AFVR and AJ, simple seasonal dynamics for AWD and ABD, and simple seasonality with irregular fluctuations for FD and NI during the study period. Prospective time series forecasting for AFVR and ABD showed a continuous increasing trend, while that for AJ showed a declining trend.

In addition, we observed a slightly increasing trend in the incidence of AFR, AFVR, ABD, and FD over the years since 2009; this may coincide with the duration of the implementation of the SSS and is likely associated with the increased distribution of data reporting and feedback at all levels during the last nine consecutive years.

AFR syndrome was the most dominant syndrome in Mongolia, at 37.7 ± 6.1 cases monthly, during the study period. Generally, this includes infectious diseases such as measles, rubella, scarlatina, erythema, typhoid, dengue fever, tick borne rickettsia, and tick borne borreliosis.

The trend in the incidence of AFR showed a low prevalence rate from 2009 to 2015 followed by a surge in the number of cases, corresponding to measles outbreaks not only in Mongolia but also in neighboring countries and others (China and South Korea) in 2015–2016 [9, 10]. Measles is a highly contagious viral disease that, despite the presence of a safe, effective and affordable vaccine that can prevent the disease for more than 40 years, remains a dominant cause of childhood death in many countries [11].

The successful implementation of the 2003 World Health Organization (WHO) Western Pacific Regional Office (WPRO) measles targets through the strengthened National Immunization Program (NIP) dramatically decreased the measles-related morbidity and mortality in 2012. However, during 2013–2015, the measles virus re-emerged in endemic countries, spreading to countries in which the measles prevalence had decreased through control [12].

In Mongolia, about 20 outbreaks had been registered since 1958 with the most recent major outbreak occurring in 2001. Mongolia was one of the four countries in the WHO Pacific region that was certified as being measles-free in July 2014. However, on March 18, 2015 the first registered case of the disease was reported in the Chingeltei District of Ulaanbaatar city, with laboratory testing showing viral genotype characteristics that were similar to those of a strain circulating in China. The outbreak was attributed to the presence of imported measles virus. The outbreak occurred in two waves, with the peak of the first wave occurring in May 2015 and the second in February 2016, with about 50,000 suspected measles cases. The number of measles-related deaths increased in the second wave, coinciding with the prevalence of seasonal ILI due to high exposure from hospitalizations during the incubation period of measles in healthcare facilities. In terms of age, the most affected populations were infants who were ineligible for the first dose of the measles vaccine at 9 months of age and those aged 18–30 years. About 74% of all measles-related deaths occurred in children aged younger than 9 months who were previously non-immunized against measles. Response measures undertaken included additional two-dose anti-measles immunization with a coverage of 88–93.4% of the target population aged younger than 35 years old; these included infants aged 0–6 months who were immunized with the “0” dose of the measles vaccine in high-density populations and measles hotspots in five provinces in Mongolia.

The incidence of AFVR syndrome, which includes illnesses such as chickenpox, and Enterovirus and Varicella zoster virus infections, showed a 31-fold increase during the study period. In Mongolia, hand, foot and mouth disease showed the highest prevalence in May 2008 with a prolonged period of presence lasting 2–3 years marked by increments in spring and decreases in autumn, peaking for 5–6 months. About 6051 cases of the syndrome were reported in 2017, indicating a surge in the number of cases compared to previous years.

AJ syndrome comprises diseases such as hepatitis A-E, leptospirosis and yellow fever. The occurrence of AJ related to a hepatitis A outbreak between 2010 and 2013, then promptly have introduced vaccine against hepatitis A virus (HAV) in the routine immunization schedule in the NIP for first dose in 9 months then second dose in 2 year olds starting in 12 provinces and Ulaanbaatar city since 2012. As a result, the reported number of HAV cases sharply dropped by 3.3 times as of October 2013 compared to the prevalence in the previous five years. In addition, more than 77% of the Mongolian population is estimated to have had hepatitis B virus (HBV) infection at some point, and between 10 and 22% of the general population has chronic HBV infection. Hence, liver cancer is the single most common cause of mortality – with one of the highest rates worldwide and six times higher than the global average [13]. In addition, hepatitis virus C treatment was introduced in 2016 while hepatitis B vaccination was expanded when entry-level workers new entering workers and other risk groups.

Early epidemic prediction is an important part of public health, since current trends are assessed to provide information on the future. Accurate and reliable forecasts with sufficient lead times may provide greater opportunities for disease control and offer effective responses to reduce the substantial morbidity and mortality related to these diseases. Early prediction can help in planning, especially during outbreaks, and provide valuable information to decision-makers and experts in other fields by indicating how far an outbreak is from being under control.

In this study, prospective time series forecasting demonstrated a potential increase in the incidence of AFVR and ABD in future years in Mongolia. Relevant specific diseases include hand, foot and mouth disease, shigellosis are like gastrointestinal diseases. Factors that may have contributed to the incidence of gastrointestinal infectious diseases include poor food and water safety, inadequate sanitation, and poor personal hygiene. Other factors include a lack of inter-sector information sharing, surveillance, response, preparedness, risk assessment, risk communication, monitoring and evaluation, and inadequacies in the number of trained workers and laboratory capacity. Further efforts should focus on the prevention of major disease outbreaks.

The use of SS has increased, worldwide, in recent years. A number of SS approaches have been proposed and the types of SS have become increasingly diverse. SS utilizes various information sources and the usage frequency of each type varies depending on each country’s medical practices. According to a US survey, 84% of the 43 public health departments conducting syndromic surveillance included surveillance for emergency department visits [14]. This was followed by surveillance for outpatient department visits (49%), surveillance for over-the-counter (OTC) medication sales (44%), and surveillance for school absenteeism (35%), as of 2007. In France, ILI surveillance is performed in 11,000 pharmacies within 21 regions across the country (about 50% of all pharmacies in France) [15]. This ILI surveillance system is a web-based system that collects medication sales and weekly office visit data to provide influenza outbreak forecasts using a Poisson regression model. In Japan, the National Institute of Infectious Diseases has developed an SSS based on the Early Aberration Reporting System syndrome categories and software to analyze OTC sales data, outpatient visit data, and ambulance transfer data in Tokyo [16]. Approximately 5000 sites nationwide in Japan are now connected to this system. In Mongolia, in the past few years, surveillance has been conducted for school absenteeism associated with acute respiratory infection in selected schools during the flu season. In addition, there is surveillance for factors such as human immunodeficiency virus (HIV) infection, tuberculosis, and immunization.

Previous studies have shown the usefulness of SS. In a previous survey study, 80 and 92% of the respondents reported that SS for influenza was “highly useful” and “somewhat useful” as compared to other methods for small outbreak detection [17]. Hence, most major countries use an SSS for ILI. For instance, SS for ILI with respiratory symptoms is conducted in Taiwan, ILI with respiratory symptoms and gastrointestinal symptoms in Britain and France, and ILI with gastrointestinal symptoms and other specific symptoms in Australia. In South Korea, 120 emergency departments from 16 provinces and cities are now connected to the Korea Emergency Department Information System for the daily analysis of acute respiratory syndrome [17]. In the US, estimated data obtained from Google Flu Trends and surveillance for prescription medication showed a strong positive correlation, and the number of searches containing the terms “influenza” or “flu” was strongly to the percentage of influenza-positive cases in clinical tests and deaths attributable to pneumonia and seasonal influenza (https://www.google.org/flutrends/about/). In Mongolia, influenza and ILI, and ARI are monitored separately by sentinel surveillance systems that include laboratory confirmation.

This study has a number of limitations. First, data were collected from weekly SSS feedback for the retrospective analysis, and due to multiple-type sources including Excel, Word and PDF files, data omission and incompleteness or the presence of errors cannot be ruled out. Data quality evaluations for the detection of reporting errors are critical for such surveillance data. Second, it was difficult to verify underreporting or over-reporting, and misdiagnosis or misclassification of the syndromic diseases in the SSS database, as data for this study included only pre-diagnostic clinical-based information without laboratory confirmation. Third, we could not consider the outbreak of hepatitis A and measles during study period for developing forecasting models of AJ and AFR. Fourth, the performance of long-term forecasting of some ARIMA models might be unreasonable. Because the forecast for AFVR seems to be too sensitive to the increasing trend near the end of 2017, we cut the forecast for AFVR at the end of 2018. Finally, we could not evaluate the accuracy of the diagnoses because we did not obtain detailed information on demographics and diagnoses through medical records.

## Conclusions

This study shows the time series trends for infectious disease syndromes in Mongolia. The surveillance system for infectious disease syndromes can be useful in providing an understanding of the current situation and forecasting the future trends of various infectious diseases. Prospective time series forecasting in the incidence of AFVR and ABD showed increasing trends, while the incidence rate for AJ showed a declining trend in Mongolia. This study provides important information on the pattern of infectious diseases in Mongolia. Further research should analyze and estimate potential forecasts using early warning signals provided for particular diseases especially during outbreaks.

## Data Availability

Apply to the availability of these data are not publicly available. Data are however available from the authors upon reasonable request and with permission of Committee. A person who wants to access the raw data should contact with the corresponding author.

## Abbreviations

ABD: Acute bloody diarrhea
AFP: Acute Flaccid paralysis
AFR: Acute fever with rash
AFVR: Acute fever with vesicular rash
AHS: Acute hemorrhagic syndrome
AJ: Acute jaundice
ALRI: Acute lower respiratory infection
ANS: Acute neurologic syndrome
ARI: Acute respiratory infection influenza and ILI
ARIMA: Auto-Regressive Integrated Moving Average
AWD: Acute watery diarrhea
ED: Emergency department
EWAR: Early Warning and Response
HAV: Hepatitis A virus
HIV: Human Immunodeficiency virus
ID: Infectious disease
ILI: Influenza like illness
NCCD: National Center for Communicable Diseases
NIP: National Immunization Program
OTC: over-the-counter
SSS: Syndrome surveillance system
WHO: World Health Organization
WPRO: Western Pacific Regional Office
